# Let Me Prep You to PREP Me: Amplifying the Voices of Black Women and Their Providers to Consider PrEP as an HIV Prevention Option

**DOI:** 10.3390/ijerph19031414

**Published:** 2022-01-27

**Authors:** Rasheeta Chandler, Dominique Guillaume, Jessica Wells, Natalie Hernandez

**Affiliations:** 1 Nell Hodgson Woodruff School of Nursing, Emory University, Atlanta, GA 30322, USA; r.d.chandler@emory.edu (R.C.); jholme3@emory.edu (J.W.); 2 School of Nursing, Johns Hopkins University, Baltimore, MD 21224, USA; 3 Community Health and Preventative Medicine, Morehouse School of Medicine, Atlanta, GA 30310, USA; nhernandez@msm.edu

**Keywords:** HIV prevention, health communication, pre-exposure prophylaxis, Black women, attitudes

## Abstract

Despite the high efficacy of pre-exposure prophylaxis (PrEP) in preventing HIV acquisition, PrEP uptake among Black cisgender women remains low. Our qualitative study assessed Black cisgender women’s perspectives, attitudes, and acceptability towards PrEP, in addition to exploring PrEP-related attitudes, facilitators, and barriers to PrEP access among health care staff. This study was conducted to ascertain data to inform the development of our HIV prevention app—*Savvy HER*—which is being designed for Black cisgender women. Our findings indicated that Black women had low levels of PrEP acceptability and high levels of misconceptions, inaccurate knowledge, and stigma towards PrEP. Health care providers in our sample confirmed barriers of stigma, misconceptions, and knowledge among their patients coupled with difficulty accessing PrEP due to structural barriers. Our study indicated that there is a critical need to heighten Black cisgender women’s PrEP knowledge and HIV risk perception in order to increase PrEP acceptability and uptake.

## 1. Introduction

Pre-exposure prophylaxis (PrEP) for HIV prevention has been integral for meeting established targets for ending the HIV epidemic [1]. Truvada (emtricitabine/tenofovir disoproxil fumarate) for PrEP, which is a pill taken daily, was approved for women in 2012, and is a highly effective medication for preventing HIV for non-HIV infected individuals. More recently, the Food and Drug Administration (FDA) approved the first long-acting injectable treatment for PrEP consisting of the drug cabotegravir, which is injected once every two months for HIV prevention [2].

In the United States (U.S.), southern states account for half of new HIV diagnoses despite only 37% of the US population residing in the South [3,4]. Atlanta, Georgia, which has a larger racial and ethnic minority population compared to other cities in the U.S., also has one of the highest HIV incidence rates in the nation [5]. Black cisgender women in Atlanta shoulder the disproportionate burden of HIV compared to their non-Black female counterparts [6]. Despite the efficacy of PrEP in reducing the risk of HIV acquisition, this HIV prevention method has not adequately been extended to Black cisgender women [1,7,8,9]. Although substantial progress has been made in reducing HIV incidence among women over the last decade, Black cisgender women are severely underrepresented among PrEP users [9]. This is highly concerning given that Black cisgender women are disproportionately affected by HIV compared to their non-Black female counterparts [1,10], with epidemiologic data indicating that Black women have a 14.6-fold higher risk of acquiring HIV compared to White women [11]. Black women experience multiple barriers that impact PrEP uptake, including low levels of PrEP knowledge and awareness, misinformation surrounding PrEP, stigma related to PrEP use, and limited access to PrEP in their geographic areas [1,10]. It is possible that the high rates of PrEP misinformation, stigma, and lack of awareness may be due to the fact that marketing for PrEP has largely focused on men who have sex with men (MSM) and transgender populations, with limited focus on Black cisgender women [10,12,13]. Due to inadequate representation of Black cisgender women in PrEP messaging campaigns, Black cisgender women report not being cognizant of PrEP as an available HIV prevention method that could potentially benefit them [7,14,15,16]. The consistent messaging gap in PrEP marketing and communication can inadvertently influence Black women’s acceptability and uptake of PrEP [12]. Furthermore, insufficient communication between patients and health care providers may limit PrEP uptake among Black cisgender women. Studies have demonstrated that PrEP prescribing tends to be low amongst primary health physicians; and even when support for PrEP among health care providers is high, the actual prescribing of PrEP remains low [17,18,19]. It is imperative to understand Black women’s perspectives about PrEP to support the development of tailored interventions that promote Black women’s PrEP uptake, as there are unique cultural, social, and behavioral influences that impact PrEP adoption in this population [19].

Additionally, exploring health care provider’s knowledge, attitudes, and perspectives towards PrEP is also imperative in meeting targets for PrEP initiation among Black women, as perceptions of PrEP among providers can influence whether Black women receive a prescription for PrEP [18,20]. HIV prevention interventions involving the use of mobile health (mHealth) technology tailored towards the needs, preferences, and lived experiences of Black cisgender women may be effective in increasing PrEP uptake in this population. However, the use of mHealth for targeted HIV prevention education and PrEP awareness for Black cisgender women have been remarkedly limited, despite Black cisgender women voicing being willing to use mHealth as a tool for HIV prevention [19].

The purpose of this descriptive qualitative study was to understand perspectives regarding acceptability towards PrEP among Black cisgender women in Atlanta, Georgia, through exploring PrEP-related knowledge and attitudes. Health care providers were also interviewed in this study to develop a deeper understanding of the factors that inhibit PrEP uptake among their Black cisgender female patients, in addition to ascertaining their perspectives in developing interventions to increase PrEP uptake and reduce HIV risks in this community. The data from this study will be used to inform the development of an mHealth app developed to provide PrEP and HIV prevention content that is tailored towards Black cisgender women.

## 2. Materials and Methods

### 2.1. Parent Study: Savvy HER

This preliminary data will inform a larger parent study to develop a mobile health (mHealth) HIV prevention application (app) for Black women, titled *Savvy HER* (Sexual/HIV Electronic Empowerment Resource) (Emory University, Atlanta, United States). *Savvy HER* will include a culturally relevant PrEP-specific health communication section, *My PrEP*, which will provide information regarding PrEP benefits, indications for use, risks, and side effects, in addition to providing up-to-date content on the latest developments for PrEP use among women (e.g., research evaluating long-acting injectables). In addition, *My PrEP* will address resource barriers to PrEP uptake through identifying PrEP proficient locals that offer PrEP for free or at a low cost. In developing *Savvy HER*, our aim was to recognize and acknowledge the disparities of PrEP uptake among Black cisgender women. The Social Cognitive Theory of Mass Communication (SCT-MC) is the theory that underpins the parent study. SCT-MC posits that critical mechanisms for behavior change can occur through mass media technology [21]. This theory is being operationalized to address health communication priorities through using media and health communication to improve sexual and reproductive health outcomes, focused on HIV prevention behaviors, while facilitating engaging, empowering, and culturally sensitive health messages.

To better inform the content development and messaging components for *Savvy HER*, we wanted to have a more comprehensive understanding of Black women’s perspectives towards PrEP. Therefore, we conducted qualitative interviews with Black women to explore their baseline knowledge, awareness, attitudes, and acceptability of PrEP. Understanding Black women’s perspectives is imperative to ensure that PrEP content is presented in a manner that is culturally relatable and relevant. Given that the *My PrEP* feature in *Savvy HER* will provide linkage to PrEP resources and clinics, health care providers were also interviewed in this study to explore barriers and facilitators to PrEP use among Black cisgender women.

### 2.2. Setting and Eligibility Criteria

Qualitative interviews were conducted with Black women who were established patients at an outpatient community-based clinic located in Atlanta, Georgia. Eligibility criteria required for women to identify as (1) Black or African American, (2) be assigned a female sex at birth, (3) be 18 years and older, (4) have no documented HIV diagnosis, and (5) currently not be on PrEP. Eligibility criteria for health care staff included (1) employed full time at the outpatient clinic and (2) employed at the clinic for at least one year. Purposive sampling was used in which recruitment occurred through flyers posted at the clinic, in addition to virtual flyers, which were sent out through the clinic’s online platforms (e.g., Facebook page and website). All participants provided informed consent prior to interviews, with Black cisgender women being compensated with a $20 gift card and health care staff being compensated with a $40 gift card at the end of the interview.

### 2.3. Study Procedures

Semi-structured in-depth interviews were conducted with participants. The interview guide was developed through an extensive literature search on the perceptions and ideations of PrEP use among Black cisgender women. Questions on the interview guide for Black women asked about PrEP knowledge and awareness (e.g., “What you have you heard about PrEP also known as Truvada?” and “What can you tell me about PrEP?”), sources of information (e.g., “Where did you hear about PrEP?”), interest in using PrEP (e.g., “Would you be interested in using PrEP if it were offered to you?”), along with benefits and harms of PrEP use (e.g., “Do you think taking PrEP for preventing HIV would be useful for you?”). Questions that were included on the semi-structured interview guide for health care providers included questions as to whether providers believed PrEP would be beneficial for their patients (e.g., “Would PrEP be a service that your patients would be willing to use?”) in addition to getting insight on providers’ personal views towards prescribing PrEP for Black women (e.g., “Tell me how you would feel in offering or prescribing PrEP in this clinical setting?”).

Interviews were conducted virtually either on Zoom or over the phone, based on the preference of participants. Zoom interviews were recorded and transcribed through the Zoom software. Phone interviews were recorded and transcribed using Otter.ai software. Following the interviews, transcripts were de-identified and edited for accuracy. After de-identification, transcripts were uploaded on Atlas.ti for thematic analysis. Two authors (R.C. and D.G.) were assigned a set of transcripts to open code, with each author coding data line-by-line and developing a list of preliminary codes. After open coding was conducted, the authors met to discuss and agree on the preliminary code list [22]. After agreement on codes was achieved, the authors finalized the list and created a code book, which was used to analyze the remaining transcripts. Codes were subsequently organized into broader categories that displayed a range within similarly coded data [22,23].

## 3. Results

### 3.1. Demographics

A total of *n* = 15 Black cisgender women participated in our study. The women’s ages ranged from 18–31 years (*M* = 25.4). Over half of participants (57%, *n* = 8) were single, and 36% of participants (*n* = 5) lived with a partner. Over half of the black women (57%, *n* = 8) were employed either full-time or part-time. A significant number of participants (57%, *n* = 8) had a form of post-secondary education (e.g., college, graduate school, or trade school) as their highest education level. Most participants had health insurance (79%; *n* = 11). Of those who were insured, 63% (*n* = 7) had a regular health care provider, while 36% (*n* = 4) did not have a regular health care provider and went to the emergency room or urgent care as needed [24]. Four health care staff were interviewed (e.g., *n* = 2 family support workers and *n* = 2 clinic providers). Family support workers are trained in providing case management alongside supportive services (e.g., linkage to resources) for patients. The staff who were interviewed all identified as Black cisgender women.

### 3.2. PrEP Awareness, Knowledge, and Acceptability—Black Cisgender Women

All of the women in our study cited condom use as their primary method for HIV prevention. Participants were asked whether they had heard of Truvada for PrEP, which is also referred to as “the blue pill”. The majority of participants confirmed having heard of PrEP. Among the women who had heard of PrEP, acceptability towards PrEP varied when they were asked if they would consider taking PrEP as an HIV prevention measure. Among the women who stated they would not be comfortable taking PrEP for HIV prevention, the most common underlying reasons were not knowing enough about the medication, concerns about side effects, and pill burden. Additional quotes are provided in Table 1.

“I am kind of scared to take pills because of the side effects…. you take this because it is supposed to help you get well, but it is actually like killing you on the inside.”—18-year-old participant who is single.

Most women were unaware that PrEP was a medication that women could use. In addition, several women had misconceptions and disclosed inaccurate information, in which they believed PrEP was only authorized for use among “gay men” or men who have sex with men. Although many women had heard of PrEP, inconsistencies were found in PrEP knowledge, as a number of women stated that although they had heard of PrEP, they were unsure of the indication for PrEP and how the medication worked.

“No [I would not take it if offered] it is just …something I would not use…at first I thought it was strictly for homosexuals.”—18-year-old participant who is six months postpartum.

Women who stated they would consider taking PrEP if offered, appeared to have more baseline knowledge on PrEP, along with having a higher awareness about Black women’s objective risk of HIV acquisition. Interestingly, the majority of women who demonstrated high knowledge and acceptability towards PrEP were either enrolled in college full-time or had a college degree.

“I know a lot of times women get [HIV] from their boyfriends or husband because he has been sleeping around so I think it would be good for someone who is in a committed relationship and they think or they know that their partner is a cheater and has cheated before. I think it would be something good for them in case they decide to continue that relationship and have unprotected sex with that person.”—30-year-old participant who is single and employed full-time.

Perceived risk towards HIV became a theme that was elicited from the interviews, as few women perceived themselves to be at risk of HIV, thus they did not believe that they would be beneficiaries of PrEP. However, some women stated that they would consider taking PrEP if they perceived themselves to be at risk for HIV. This finding indicates the role of perceived risk in potentially influencing PrEP uptake.

“Mmm I mean if I considered myself high risk [for HIV] then possibly [ I would consider using PrEP], but I am pretty low risk I believe um I am a one partner type of person and I pretty much have been in a long-term monogamous relationship…”—27-year-old woman who is 4 weeks postpartum.

### 3.3. Sources of Information and Lack of Representation—Black Cisgender Women

Participants cited various sources of information where they heard about PrEP, which included social media, television commercials, billboards, peers, and health care providers. Participants who stated they had heard about PrEP through advertisements were asked about their perceptions towards the marketing and advertising for PrEP, and whether they viewed advertisements as being relevant towards their needs and experiences as a Black woman (Table 2).

“When I see the commercial that is why I said I did not know if I needed to have it [PrEP]… because the commercial kind of gives that [impression], you know, I believe it is a transgender then it is a gay male commercial. So, it is showing the [who] the commercials geared towards. So that is why I was like, ‘do I have to have HIV to get it?’ Or is it ‘No, I do not need to have HIV and I can take it.’…the commercial does give the impression that it is not for the heterosexual community.”—24-year-old woman who is living with their partner.

### 3.4. Perceptions of PrEP—Healthcare Staff

Health care workers had varied insights regarding whether their Black cisgender female patients would be receptive to PrEP uptake. One health care provider stated that they believed acceptability of PrEP if offered would be low, given her experiences in providing HIV and other STI screenings for patients at the outpatient clinic, in addition to her past experiences working with predominantly Black patients in other clinical settings in metro-Atlanta. Other health care providers and staff, however, stated that they believed their patients would demonstrate interest in PrEP if they were offered the medication. Family support workers specifically highlighted difficulties in getting their patients to use condoms, and therefore stated that offering PrEP would impose further challenges.

“No [clients would not be interested in taking PrEP] based on [my experience] completing STD screening, which includes an evaluation for PrEP qualifications. They meet the qualifications for PrEP [uptake]. Some females take it but not that many. I would say, out of my years working here, every guy has declined PrEP, unless they were an MSM, and as for the females, the majority of them did not want it.”—Health Care Provider.

### 3.5. Barriers to PrEP Use—Healthcare Staff

Barriers to PrEP use for patients was an emerging theme among interviews with health care staff. The barriers that health care staff cited were categorized into broader themes which included patient barriers (e.g., high cost of PrEP, misconceptions, and stigma), structural barriers (e.g., inadequate health care infrastructure and lack of clinic resources), and barriers among health care providers in prescribing PrEP (e.g., discomfort in managing patients on PrEP). Clinic providers specifically stated that they did not see the benefits of prescribing PrEP, if their patients would have tremendous difficulty in accessing the medication due to cost.

“PrEP sounds great, but when the client calls me in need, sure I can write your prescription but is $3000 per month worth it? or I do not know if it is that much but you know, thousands of dollars. That is not feasible, so if we had a program where to fund the medication that would be great.”—Health Care Provider.

Healthcare staff stated that their patients had significant misconceptions and stigma surrounding HIV and PrEP, which limited the uptake of PrEP among Black cisgender women. The primary misconception towards PrEP included Black women viewing PrEP as a drug only used among gay men. Health care staff also mentioned hesitancy towards PrEP use due to pill burden. The barriers to PrEP use that was cited among health care staff mirrored what was noted during interviews with Black cisgender women.

“I have clients that are now naturalists and think birth control is bad and condoms knock off your pH balance and things like that. I do always talk about [PrEP] and most of the time they have not heard of it or they think it is for gay people…well gay men. So, I have to let them know that it prevents HIV.”—Health Care Provider.

Health care providers stated being uncomfortable prescribing and managing patients on PrEP due to lack of knowledge, insufficient clinic infrastructure for PrEP efforts, and shortages of human resources to adequately follow-up with PrEP patients. Statements were made about difficulties for ensuring clients were adherent with taking their birth control pills daily, and that, as a result, there may be challenges for ensuring that patients are adherent with taking daily PrEP if prescribed.

“I just wish there was a better infrastructure at the clinic to offer PrEP …I feel bad offering women PrEP and then sending them elsewhere to get PrEP….”—Family Support Worker.

## 4. Discussion

Our qualitative study explored perspectives regarding PrEP knowledge, acceptability, and feasibility through identifying barriers and facilitators to PrEP use among Black cisgender women and health care staff in Atlanta, Georgia. The findings from this study are important to consider, given that Truvada for PrEP has been approved since 2012, however the uptake of Truvada among Black cisgender women has remained consistently low, despite their high HIV risk. Given the overrepresentation of Black women in new HIV diagnoses, understanding their perspectives towards PrEP is critical in identifying the factors that may influence PrEP uptake [12]. Additionally, as more PrEP options are developed and approved for cisgender women (e.g., long-acting injectables), understanding the key barriers and facilitators towards PrEP acceptability and uptake will be critical in increasing the use of new PrEP modalities and improving Black cisgender women’s sexual health and wellness.

Black women in our study demonstrated high PrEP awareness, with many stating they heard about PrEP through various information sources. However, the majority of participants had inadequate knowledge on PrEP and had misconceptions regarding PrEP. These findings mirror similar results in studies that have assessed PrEP acceptability and uptake among Black cisgender women in the U.S. [12,24,25]. Low PrEP knowledge and high levels of misinformation and HIV stigma have been documented as barriers to PrEP among Black cisgender women [10,12,19,26,27,28,29]. Among our participants, the primary misconception was that PrEP was only preventive for individuals in the LGBTQ community, with certain participants stating that they perceived PrEP to be a “gay drug”. These statements were confirmed by the health care staff, who stated that many of their Black cisgender patients believed PrEP was strictly for gay men. These misconceptions may be largely reflective of current marketing campaigns for PrEP, which primarily target LGBTQ communities, while not effectively targeting Black cisgender women, despite their high HIV risk [1]. The lack of representation of Black cisgender women in PrEP communication and marketing campaigns has been reported in several studies, and reflects the need for more targeted PrEP messaging tied to Black women’s perceptions of who is at risk of acquiring HIV, with appropriate representations of Black cisgender women [1,10,15]. There was triangulation between the responses from health care staff and Black women, which confirmed that Black women had relatively low acceptability towards PrEP. This was further compounded by the heightened misinformation and stigma surrounding PrEP.

Among the few women who demonstrated higher acceptability towards PrEP, the majority had higher PrEP knowledge levels, and had received recent education on PrEP prior to participating in the study. This is critical to note as this may indicate that comprehensive PrEP education functions as a key facilitator in increasing PrEP acceptability and potentially PrEP uptake, which has been noted in previous studies [10,24,30,31]. Thus, the incorporation of PrEP education and referral to health care providers who are knowledgeable on PrEP must be considered in the design of HIV prevention interventions for Black cisgender women [24].

The majority of women in our sample were not willing to use PrEP, with certain women stating that they would only consider PrEP if they felt themselves to be at risk of acquiring HIV. This may reflect a lower risk perception towards HIV in our sample. It has been found that individuals who would be considered to be at high risk of HIV based on objective risk assessments often perceive their own HIV risk to be low [32].While all women in our study cited condom use as a method of HIV prevention, the responses from the health care providers in our study highlighted that not all their patients were engaging in regular condom use. This finding may also be indicative of low HIV risk perception. Due to low rates of condom use among patients, health care staff stated that promoting PrEP for their patients may prove difficult. However, it is important to emphasize that condom use requires women to negotiate with partners, or otherwise rely on partner-initiated behaviors. Given that PrEP is self-directed and can be administered without partner awareness in many cases, it may offer a better prevention strategy, in which women are able to take immediate control of their sexual health [33,34]. Therefore, it is important for health care providers and staff to provide comprehensive education that promotes the benefits of PrEP from a women’s empowerment and sex positivity perspective. Health care staff who stated that PrEP would be beneficial for their patients noted the importance of informing patients about the benefits of PrEP and ensuring that patients have appropriate information to make informed decisions.

Discomfort in managing patients on PrEP was voiced by one health care provider in our study. Furthermore, one health care provider also stated that, given their previous experiences, they did not think PrEP would be beneficial for their patient population. This opinion was also reflected among family support workers. Reluctance in prescribing PrEP coupled with bias in PrEP prescribing has been noted among health care providers in previous studies [35,36,37]. There is a need to ensure that Black cisgender women are linked to the adequate resources they need to obtain PrEP. These resources include access to health care providers who are knowledgeable in and comfortable prescribing PrEP. A significant concern that limited health care provider’s abilities to effectively link patients to PrEP were cost-related factors. PrEP costs have been a documented barrier to PrEP access in numerous studies [25,32,38,39]. This is important to consider as public health interventions must ensure the accessibility and sustainability of PrEP. If PrEP education is provided to individuals, they should be provided with the necessary resources to access PrEP at a low-cost. Truvada, which previously cost upwards of $1800 a month, has recently gone generic, with federal mandates requiring health insurance companies to cover all costs for PrEP treatment [40,41]. This may alleviate significant financial barriers for individuals who are insured [38,39], however access may be a burden to individuals who are uninsured. Manufacturer’s patient assistance programs (e.g., Gilead Advancing Access) should be considered for patients who are uninsured, however health care providers must be aware that these options exist for patients [30].

### PrEP Savvy Features for Promotion of PrEP Uptake

The data obtained from this study, along with previous studies conducted by the research team, will be used to inform the development of the *My PrEP* feature in the *Savvy HER* mobile app, which will include comprehensive PrEP information coupled with targeted and adaptive messaging [42,43]. Targeted messaging for Black women involving the diffusion of information through content-specific communication via mass media campaigns can be impactful in addressing barriers to PrEP uptake. Mass media and messaging campaigns have demonstrated efficacy in HIV prevention and health promotion for Black women outside of the United States, with a number of studies demonstrating a relationship between exposure to HIV mass communication programs and higher HIV knowledge levels, less stigmatization towards HIV, and increased likelihood of HIV prevention behaviors (e.g., HIV screening and condom usage) [44,45,46,47]. Given the findings from our study, coupled with the lack of interventions focused on developing targeted PrEP-related health communication for Black women, the *My PrEP* feature in the *Savvy HER* app will be developed to include targeted health messaging consisting of various multimedia methods (e.g., video clips, infographics, and audio) focused on PrEP for Black women.

Most women in our sample were unwilling to use PrEP, with certain women stating that they would only consider PrEP if they felt themselves to be at risk of acquiring HIV. This may be indicative of a lower risk perception towards HIV. Several participant responses indicated that they were in a monogamous relationship with a committed partner or had not had sex in years. However, studies have noted that Black women may be less likely to use condoms in monogamous, committed relationships compared to casual and multi-partner relationships [48,49]. It has been suggested that women in such relationships have decreased perceptions of HIV risk, resulting in decreased willingness to use PrEP and decreased condom use [48,49]. Consequently, this can inadvertently place women at risk for HIV acquisition, especially among women who are in relationships with men who are non-monogamous [50]. Given the low HIV risk perception demonstrated among our participants, in addition to similar findings being reported among Black cisgender women in other studies, *My PrEP* will include adaptive health messaging that will employ personalized messages, to both combat dampened risk perception and adverse attitudes towards PrEP uptake. Through the app, end-users will have the ability to log their sexual and reproductive health data, which will generate an interactive dashboard to help women identify meaningful trends in their tracked data. This tracked data will be used to generate tailored adaptive HIV prevention and reproductive health messaging that is customized for each woman based on the information they input. The app will aggregate data on behaviors, attitudes, facilitators, barriers, and needs that are relevant to Black women’s sexual health, while also being personalized at the level of the individual, which will inform the development of prevention messaging. Our findings demonstrate that ensuring that Black women have adequate PrEP and HIV-related knowledge is important to foster PrEP uptake, however it is also essential to ensure that Black women have access to appropriate resources to successfully obtain PrEP. *Savvy HER* will include a linkage to resources feature to increase PrEP accessibility for Black cisgender women. We will use the feedback obtained from women and health care personnel in our study in addition to data from prior studies conducted by the research team to inform the development of the resource tool to assist Black women in accessing PrEP [41,42]. The *Savvy HER* app will also include a feature that links end-users to health care providers for HIV prevention information and sexual and reproductive health services. The cumulation of these features may significantly strengthen HIV prevention and PrEP communication between Black cisgender women and health care providers, while also influencing HIV prevention communication among sexual partners.

## 5. Conclusions

There has been a scarcity of literature fully evaluating and exploring Black cisgender women’s perspectives towards PrEP, in addition to limited studies exploring barriers and facilitators to PrEP use for this population from the perspectives of health care personnel. Our study indicated that there is a critical need to heighten Black cisgender women’s PrEP knowledge and HIV risk perception in order to increase PrEP acceptability and uptake. Health communication and messaging interventions tailored for Black cisgender women that target misconceptions, stigma, attitudes, and risk perception may be significant in increasing PrEP acceptability and uptake in this population. Furthermore, there is also a need to address additional structural challenges within the health care system that may pose challenges in PrEP access for this high-priority population. Our findings substantiate the need for the development of tailored strategies to increase PrEP acceptability and uptake, such as *Savvy HER*, for Black women, particularly as the women in our study were residing in a high-burden area for HIV. It is imperative to ensure Black women are empowered with the correct knowledge and provided with the resources needed to engage in PrEP for HIV prevention.

### Limitations

Our study had a small sample of *n* = 15 Black cisgender women and *n* = 4 clinic staff. As a result, our findings cannot be generalized to the broader population. However, given that this was a descriptive qualitative study, our aim was not to draw broad population-based inferences, but to instead elicit rich data on the factors that influence PrEP uptake from the perspectives of Black cisgender women and the health care staff who work directly with this population. We enhanced the credibility of our findings through the triangulation of data between Black cisgender women and health care staff.

## Figures and Tables

**Table 1:** Additional quotes from Black cisgender women.

Domain	Theme	Quote	Participant Characteristics
Barriers to PrEP uptake			
	Low acceptability	“Um, no [I would not take it if offered] and the only reason why I say that is because I do not know enough about the medication.”	24-year-old participant in a relationship
	Risk perception	“It is not really something I have thought about…because I do not have sex….I think it has been like four years for me.”	28 year-old single woman who is employed full time
	Side effect concerns	“I am kind of scared to take pills because of the side effects…. you take this because it is supposed to help you get well, but it is actually like killing you on the inside.”	18-year-old participant who is single
	Low Knowledge	“How does that pill work? is that for people who have an STD?”	18-year-old participant who is single and sexually active
Facilitators to PrEP uptake			
	High Knowledge	“I actually did a report on it [PrEP] yesterday for my class, and I feel that the way that it is presented is actually very good and it is accurate… if it were to be offered, if that was something that I needed, I would definitely use it.”	18-year-old participant who is in college full-time
	High acceptability	“Yes, I would use it…I must say it is definitely used for HIV, which is common among in Black women, so I think that would be good for a woman to protect herself.”	19-year-old participant who is single and is a full-time college student
	Risk perception	“If I felt like I was being intimate with someone who had HIV, then yes I would most definitely want to take the pill.”	22-year-old woman who is living with their partner
Representation of PrEP in the Media			
	LGBTQ Drug	“The way that they present [PrEP] is more as a LGBT type drug and it automatically makes someone who is heterosexual say no I do not need that, it is not for me.”	24-year-old single and employed full-time
	Promiscuity	“With the commercials I think it is something good for people who are promiscuous, or I guess free spirited …something good for them to use...people who have more [partners] or who do different stuff…”	30-year-old woman who is single and employed full-time

**Table 2:** Additional quotes from health care staff.

Domain	Theme	Quote	Participant Characteristics
Patient barriers to PrEP uptake			
	Low Patient Acceptability	“Well, when we first offered PrEP in [another Atlanta-based clinic] the manager told us ‘Hey, we will have this talk [with patients] about it’. [However]. They are not super, excited about even discussing [PrEP]. I can barely get women to use condoms.”	Family Support Worker
	Challenges in uptake of sexual and reproductive health prevention methods	“Black women are a group we want to help make sure we provide sensitive care to, but at the same time, women do not use condoms very much…so to be like ‘hey do you want to use PrEP’ I feel like that is another hurdle [for sexual health prevention]. I have a hard time getting women to use contraception.”	Family Support Worker
	Cost Barriers	“If PrEP were offered at no cost that would be great. But definitely at a reduced price…it is expensive. I forgot how much…but it was a lot…so um, yeah for free. It would be very helpful for the ladies.”	Health Care Provider
	Stigma	“The race and stigma behind PrEP, alongside keeping clients interested is something [to consider].”	Health Care Provider
	LGBTQ Drug	“PrEP would be very helpful for the ladies. Outside of the price being a barrier, yeah, the stigma behind PrEP is a barrier… it seen as a ‘gay man’s pill’…”	Health Care Provider
Provider barriers to providing PrEP			
	Discomfort managing patients on PrEP	“I myself am kind of hesitant to manage a patient on PrEP.”	Health Care Provider
	Costs	“I know PrEP is expensive if it was at a reduced price or no cost that would be great.”	Family Support Worker
Facilitators to PrEP uptake			
	Provider acceptability	“Yeah. So, I think this population would highly benefit from it. I think it is a myth to kind of think that this population is scared of it. I think we inform these patients of the benefits of it and have them make the decision”	Family Support Worker
	High Patient acceptability	“Honestly, we probably have more interest [women] in starting and maintaining PrEP than those who are not interested.”	Family Support Worker

## Data Availability

The data presented in this study are available upon request from the corresponding author. The data are not publicly available to maintain confidentiality among participants who were interviewed.
